# “I have some wishes, which are actually demands.” A qualitative mixed methods study on the impact of consumerism on the therapeutic relationship in mental healthcare

**DOI:** 10.3389/frhs.2024.1388906

**Published:** 2024-11-22

**Authors:** L. T. E. Krikken Mulders, E. H. Tonkens, M. J. Trappenburg

**Affiliations:** ^1^ Citizenship and Humanization of the Public Sector, University of Humanistic Studies, Utrecht, Netherlands; ^2^ School of Governance, Utrecht University, Utrecht, Netherlands

**Keywords:** consumerism, mental health care, therapeutic relationship, patient narratives, professionalism, logic of care

## Abstract

**Introduction:**

Alongside the logic of care, many Western welfare states have introduced market elements or a logic of choice in their healthcare systems, which has led to consumerist behavior in patients. For the medical field, it is well documented how consumerism creates complex ethical dilemmas and undermines ways of thinking and acting crucial to healthcare. Little is known about these dynamics in mental healthcare.

**Methods:**

This study used a qualitative mixed methods design, combining 180 online patient narratives (blogs) with 25 interviews with therapists in a grounded theory approach.

**Results:**

Findings show that articulate behavior can be divided into two categories: assertive and adamant. While assertive behavior is understood as an integral, reciprocal part of therapy and is stimulated by therapists, adamant or consumerist behavior is experienced as damaging the relationship—the “commodity” the patient is seeking to obtain, as the single most important predictor of treatment success. Findings also show that articulate behavior in both varieties takes a different shape over time during the course of treatment.

**Discussion:**

Adamant behavior clashes with the internal logic of care, which is especially problematic in mental healthcare where the relationship with one's therapist is key to successful treatment. Therefore, patients should be taught and helped to display assertive behavior without resorting to adamancy. Individual therapists cannot achieve this alone; this endeavour should be supported by their organizations, societal beliefs about therapy and policy choices.

## 1 Introduction

In 2008, anthropologist and philosopher Annemarie Mol published *The Logic of Care* (1). Mol argued that two different logics operate in healthcare: the “logic of care” and the “logic of choice”. Within the logic of care patients are just that: patients that need proper care and attention. However, they are not passive partners in their contacts with clinicians, as many diseases require a lot of patient work. Mol studied diabetic patients who needed to mind their daily diet, measure their sugar level, self-inject insulin and more. Nor are they simply following doctors’ orders: since the democratization of health care, patients and doctors discuss the best way forward to fight or treat the disease or make it manageable in daily life.

In the logic of choice, on the other hand, patients feature as consumers who buy products after having studied advertisements and customer reviews. Montori (2) describes how, since the advent of neoliberalism, patients have been admonished to behave as consumers while their clinicians are supposed to operate as entrepreneurs. Some researchers are optimistic about the impact of the logic of choice, arguing that introducing market elements in the public sector might lead to efficiency (speedy and cost-effective service because customers might go elsewhere), transparency (customers need to be able to choose, hence service providers should be open about what they can and cannot deliver) and democracy (elevating the dependent party to a customer who can demand proper service) (3, 4).

However, both Mol and Montori argue that the logic of choice is not suitable for a healthcare system. Montori poignantly describes how: “Patients, buying into the idea that they are consumers in a marketplace in which you must ‘ask your doctor about …’ show up demanding care that they don't need. Fulfilling these requests reinforced by financial rewards linked to satisfaction surveys, offends the clinician's sense of what is right for the patient.” Montori's observation that consumerist behavior can create complex ethical dilemmas for the professional and undermine ways of thinking and acting crucial to healthcare, has been found in other studies as well. Patients might insist on unnecessary tests, treatments or referrals (5), refuse care that would be best according to clinical guidelines (6) or be reluctant to relinquish ideas about health and illness that are unrealistic or undesirable from a medical perspective (7), sometimes found on the internet (8).

Although the logic of choice might sound desirable from a patient's point of view, research has found that executing it requires skills and resources many patients don't possess. The ideal of informed health care consumers who can advocate their own interests at any given time, is often a far-fetched myth, due to the fear and stress of being ill and insufficient knowledge (9–11). As a result, even if patients articulate concerns, they tend to do so implicitly by hinting at unpleasant emotions, rather than clearly and unambiguously stating them (12).

In the last chapter of her book Mol maintains that the two logics may play out differently in different parts of healthcare. Montori also uses examples from diabetic care and builds on other parts of somatic care, mostly oncology. In this paper we want to find out how the logic of choice plays out in mental health care.

A few studies discuss the logic of choice in mental healthcare (13–15) but its scope, expression and impact are not well documented. In this article we focus on the therapeutic relationship (thus leaving aside other aspects of marketization), which is deemed the most important predictor of treatment success in mental healthcare (16, 17). Previous research suggests that psychotherapists can cope with very difficult behavior from clients. They are accustomed to working with a conflicted patient population (18), consider the negotiation of expectations and treatment goals a normal part of their work (19, 20) and are trained to mobilize tension in the therapeutic alliance as an opportunity to help patients overcome dysfunctional interpersonal patterns (21, 22).

However, individual clinicians suggest that consumerist behavior belongs to a class of its own, is particularly difficult to reconcile with the reality of psychotherapy and cannot be influenced sufficiently through trained therapeutic techniques and skills [e.g., (23–26)]. These are clinical observations, not outcomes of academic research. We therefore lack systematic insights into the manifestation of the logic of choice in the consultation room. To gain insight into this phenomenon we will investigate patients’ and therapists’ perspectives.

## 2 Material and methods

Apart from the concepts “logic of care”, “logic of choice” and “consumerism” which inspired our research question “how does consumerism play out in mental healthcare” we used a bottom-up mixed methods design, building theory from qualitative data using the methodological framework of grounded theory (27–29). We used a sequential design which will be described here in the order in which it took place. For our analysis of what patients and therapists were communicating and why, we combined our expertise in psychology (first author), sociology (second author) and governance (third author). Our chair group focuses its research on the development of citizenship in its many facets, supported by a public sector based on dignity, fairness and humanity.

### 2.1 Procedure and analysis

#### 2.1.1 Patients’ accounts

To probe patients’ perspectives we studied online narratives, or blogs. Blogs have grown in popularity as they provide a dedicated free space to create public accounts of private opinions and experiences (30–32). Analysis of these accounts provides insights into the lives of people with mental health issues that hitherto remained hidden (33, 34). Blogs offer data in a naturalistic form (35), composed without the expectations of a researcher in mind (36). By using texts that were publicly available, the researchers also avoided taking up patients’ time and energy.

Using Google's search engine, we found 7 group blogs. The blogs contained 84 individual posts where patients with a wide variety of psychological symptoms described their experiences in various mental healthcare settings. We included Dutch blogs published between August 2019 and August 2020, which described or reflected on an interaction in mental health care where a psychologist was involved. One group blog was eventually excluded because it didn't meet those criteria.

We open-coded these blogs using NVivo. In total, 547 fragments were coded, distributed over 149 codes which were found inductively. Towards the end of the selection, data saturation was reached: no new codes were needed to label fragments in the data. Codes with a very small (<3) number of fragments assigned to it were reconsidered and mostly merged with other categories. Codes with an unusually high (>20) number of fragments were reanalyzed and split up into more specific categories. All codes were evaluated in terms of meaningfulness and (hierarchical) relationship with other codes. Eventually, 78 codes remained, summarized in a preliminary code tree. Memos were kept to capture ideas about meaningful ways to organize and understand the data.

In this round, we quickly discovered that consumerism did not fit all utterings of patients on their blogs, as shown by one motivated patient in the following:

I am rejected everywhere! Why? Too complex, afraid to start treatment because of my cardiac problems, not the right place for me etc. This is what makes me so incredibly sad. I want help SO badly, I want to get better. But no one has the guts to treat me. Not even the largest trauma centers. (…) This is health care in the Netherlands. People who need help so badly but aren’t accepted anywhere. Someone should stand up and do something. Today that person is me. I write this blog hoping it will reach people who can change it, like mental health care professionals, in trauma centers, or the government etc. [02-06]

This patient doesn't seem to feel she is in the position of a customer, and the mission she sets for herself is not that of an angry consumer (who would possibly say they are entitled to care); it's one of a desperate patient (who describes needing care badly). However, she does explicitly articulate a position and a goal here. We decided to deviate from the terminology of consumerism to describe and interpret what happens in mental healthcare. We use the term “articulate behavior” for all utterings of patients which referred to “the (right to) recognition as an adult and a qualified participator, or to the wish or ability to decide, act and evaluate independently” [definition of the Dutch word “mondigheid” in Thesaurus Zorg en Welzijn (37)] We found that articulate behavior comes in two varieties, which we refer to as “assertive” and “adamant”. We will explain what these terms entail in the Results section. We make this distinction based on the nature of the behavior and the internal processes that precede and follow it. Also, we found that articulate behavior took a different shape in different stages of the therapy. We related this to shifting levels of health literacy and psychological distress. As people became more acquainted with the world of mental health care, this opened up new ways of making decisions, acting and evaluating their care. For instance, the blogs provided quotes that clearly indicated that patients now knew what to ask for and who and how to ask. Many quotes indicated that patients grew towards a more equal position in the therapeutic dyad. Not being as distressed as they were in the beginning also seemed to support this process.

To find out more about the impact of the stages of therapy we studied 46 blogs found via the first round blogs, where several entries by the same authors were included over time (3 months up to a year). Cues from the bloggers indicated in which stage of therapy they were, for example, one patient described an intake session (early stages), another described some experience in the mental health care field and an existing therapeutic relationship, but listed many goals she still had to work on and predicted needing help for a long time to come (middle stages), and a third reflected on years of several different therapies and currently only needing follow-up sessions to maintain results and prevent relapse (later stages).We used axial coding for these blogs, focusing on the two varieties of articulate behavior—assertive behavior and adamant behavior—and on the stages of therapy found in the first round. During this round of analysis, special attention was paid to the context, conditions, interactions and consequences of consumerist or assertive behavior (29). In this phase, 427 fragments were coded, distributed over 161 codes, after which data saturation was reached. Some of the former categories disappeared or were merged, especially those concerning emotions, and categories describing background, behavior and results were added (and sometimes later merged when deemed to specific). Eventually, 98 categories remained and were described in a code book. Hierarchies between the categories were specified.

Subsequently we set out to test the theory built in the previous rounds by studying 50 new blogs, found by another open Google search. This prevented possible distortion of the results if group blog moderators have an agenda in promoting or withholding narratives of a certain kind (round 1) or by relying too much on the experiences of a limited number of people (round 2).

The new narratives were used to further describe and flesh out the core concepts, and come to a coherent story in which the relationships between concepts became apparent.

We paid attention to the quality and validation of the results, as well as our own theoretical sensitivity, by gaining insight by reading and discussing the blogs and their content (38), by using Seale's (39) checklist with regard to qualitative data analysis and by reflecting on our role, by systematically recording shifts in perspective at various times throughout the analysis (40).

For instance, a quote like the one below can be read and interpreted in different ways:

If you do decide to quit eventually, it is important to discuss this thoroughly with your therapist. At what pace will you decrease the frequency of your sessions, how do you want to say goodbye, and do you have any last goals you want to work on? What will you do if you're not doing well anymore and you are no longer in therapy? Are there any other things you want to discuss? Go over this together and speak up if things are moving too fast. [01-02]

There is a hint of one-sided, possibly consumerist action here (*you* decide to quit and negotiate the preconditions under which *you* can do so), but the call to action from one patient to another is to “go over this together”. The imperative to “discuss this thoroughly” and the example questions the writer gives—she writes on a peer counselling blog—speaks of increased health literacy and the normalization of asserting oneself in the therapeutic relationship, while respecting the opinion and position of the therapist. The context of the blog, which aims to help patients navigate mental health care and has a constructive, non-strident tone, is also important here. This writer gives examples from her own therapy and how her therapist responded positively and sensitively to behavior as described above. As such, it is a rich example of an assertive response during the later stages of therapy.

Table 1 presents an overview of our blog study into patients’ experiences.

**Table 1:** Analysis of online patients’ narratives.

	First round	Second round	Third round
Number of blog posts	84 blogs	46 (new) blogs by six authors	50 (new) blogs
Sampling method	Google search	Snowball sampling from blogs in first round.	Google search
Method	Open coding	Axial coding	Selective coding
Focus	A wide variety of experiences, to get a first idea of wha's going on in the field.	Descriptions of a limited number of writers over time.	Testing ideas against new material. Open search to identify new patients.
Inclusion criteria	Publication on a Dutch patient group blog concerning mental health. Published between August 2019 and August 2020 Described or reflected on an interaction in the mental health care field, where a psychologist was involved. Tags: therapy, help, recovery, healing, treatment, and psychologist were used.	Publication by patients on their individual websites ^a^[with the exception of one patient who wrote (unedited, open access) blogs on the institutio's website]; Published between August 2019 and August 2020; Described a therapeutic interaction as a scene OR; Reflected on a specific aspect of their experience as a patient.	Popped up on “blog experiences mental health care”, “blog experiences therapy”, on the first 10 results pages; Did't belong to a commercial organization, a registered association or a provider of mental health care^b^ Not written from a professional's perspective; Described a therapeutic interaction as a scene OR; Reflected on a specific aspect of their experience as a patient.
End marker	Data saturation	Data saturation	Data saturation
Adjustments	Merging small categories (<3 codes), splitting up large categories. (>20 codes)	Adjusting categories.	Adoption of certain categories as theoretical concepts and to develop a theoretical model.
Results	Preliminary code tree and memos.	Adjusted code tree and memos	Final code tree, theoretical concepts, theoretical model, memos.

^a^ Attention was shifted to individual websites to retrieve more entries from the same writer, in order to better understand articulate behavior in different stages of therapy.

^b^ This wasn't an exclusion criterion in earlier rounds because group and personal blogs by definition don't belong to a commercial organization.

#### 2.1.2 Therapists’ perspectives

We then decided to find out whether our findings regarding articulate patient behavior during various stages of therapy was recognized by therapists.

We draw on an existing dataset consisting of 25 semi-structured qualitative interviews with mental health care workers, held in 2020 and 2021 by the first researcher, a trained psychologist herself. To promote uniformity in the sample, only professionals were included who conduct therapy (i.e., psychologists) as opposed to professionals providing other interactions with patients (e.g., prescribing medication, providing practical support). All therapists were practicing, licensed psychologists with extended clinical training, who worked in mental health care and provide care under the health insurance law. These criteria ensured that all participants had sufficient experience and a general lived understanding of problems in the field (years of experience: mean 20, median 18, mode 9). The criteria also excluded other settings that are governed by different policies, such as prisons or hospitals.

As the interviews were performed for a broader study on different aspects that may put pressure on professionals, including not only patient behavior but also as policies and organizational culture, the interviews addressed more topics than necessary for this paper. For this paper, we focused on what therapists expressed about patient behavior, including their responses to follow-up questions about their emotions, their ideas about how and why such situations had come to exist, and case descriptions where professionals felt they were under more pressure than usual, with follow-up question focusing mainly on coping strategies and moral decision making. To reduce the potential for selection, confirmation, or potential “halo” effect biases, questions were phrased in such a way that they directly addressed the experience, for instance by asking for examples of specific observations or reactions (41).

Respondents were found through snowball sampling, by starting from the network of the first author and asking participants to identify other respondents who might be interested in being interviewed. Participants received information about the aim of the interview and signed a consent form adapted to interviewing. The research proposal and data management plan were reviewed and approved by the ethical committee of the University of Humanistic Studies. Data are treated confidentially and stored in a secure location. The names of participants were removed.

The interviews were transcribed verbatim and coded through an inductive process, using NVivo. For this article we selected and coded all interview fragments pertaining to articulate patient behavior using the codes and concepts developed in the previous three stages of the research.

Subsequently we combined the data from the blogs and the interviews. This deserves some explanation. Combining similar data—either blogs or interviews with both groups—would have been optimal. However, therapists don't maintain blogs comparable to those of patients, because they have the professional obligation of confidentiality which patients do not have. Interviews with patients, conversely, are difficult to obtain because of the same therapeutic confidentiality (therapists will not provide a name of possible respondents to be interviewed). Nevertheless, combining interviews and blogs demands some reflection on their differences and similarities. Qualitative interviews usually show a high degree of self-reflexivity, induced by the interviewer. Blogs, conversely, cannot be influenced by the researcher. If we would have compared blogs on, for example, shopping experiences with interviews with salespersons, this would probably create a problematic disbalance. However, in the case of patient blogs, self-reflection seemed rather common, because people write about a setting -therapy- where self-reflection is a core activity. Therefore, blogs by patients and interviews with therapists could very well be combined, as they gave different perspectives on the same types of experiences and reflected on their roles in these in a comparable fashion.

## 3 Results

In this section we will present our findings using both datasets. That is, we present the theory we developed using both the interviews with the therapists (referred to as TI and then numbered) and the patient blogs (referred to as PB and then numbered; some blogs are large and contain entries written by different patients, hence the same numbers may occur more often than customary in qualitative studies using interviews). The interview fragments and the blogs are not related, hence the therapists discussed in the blogs are not the ones interviewed here and vice versa: the patients discussed by the therapists are not the ones who contributed to the blogs (an extremely unlikely coincidence aside).

As described under “procedure and analysis”, we found two strategies or pathways for articulate behavior. The first was *assertive* behavior, which we defined as the act of asserting a right, while respecting the rights and dignity of the other person, usually preceded by internal processes which create the foundation for the response, such as perspective taking, self-regulation, and assessing the situation [based on the work of (42)]. For the second pathway we use the term adamant, which the Cambrige Dictionary defines as “impossible to persuade, or unwilling to change an opinion or decision” (43). We built on the dictionary definition because the word “adamant” is uncommon in literature on adult behavior in social situations and on patient behavior. As we will see, adamancy can be related to consumerism [as in the patient quote: “The health care consumer does not receive the guidance and treatment he needs.” (02-05)] but it does't have to be, as in the example below. Notice that, like the patient cited above, this one is also using the word “need” instead of market-based language such as “want”, “am looking for” or “am entitled to”.

But the tension rises. I know I am not the ideal patient. I am not happily cooperating and I don't care about these f***ing goals. Or course, I had signed the treatment plan. But to me it was a formality, it opened the doors to therapy. For too long I had wandered around various departments and every time after a few weeks or months there was that conversation where they told me they could offer me what I needed. I just didn't want that to happen again. [01-01]

Before embarking on the topic of articulate behavior, it seems good to remark that the general tone of the blogs was positive. We observed a consensus among patients that although the therapeutic process is difficult, it was worthwhile in the end and marked a positive turning point in their lives. Most people were content and expressed gratitude. They often emphasized the therapeutic connection, the way they got to know themselves better and general feelings of strength, rather than a reduction in symptoms. Articulate expressions were often preceded by prompts, like a change in emotional stage, a problem with the system, aspects related to the therapist or the therapy, or secondary circumstances. Sometimes, an articulate response was expected but not given. Patients expressed several obstacles that prohibited them from asserting themselves, such as being afraid of the answer, not knowing how, feeling the desire to be a “good patient” and awareness that one is using “scarce goods”. Examples are presented in Table 2.

**Table 2:** Prompts and obstacles for articulate responses.

Prompts for articulation		Obstacles for articulation	
Prompt	Examples	Obstacle	Examples
Emotional state	Embarrassment, discomfort, anger.	Afraid of the answer	Expectation that requests will not be fulfilled anyway; no options.
System	Protocols, diagnoses, questionnaires, departments, waiting lists.	Don't know how	Need more time to process, lack of communication skills.
Therapist or therapy	Mismatch in expectations, confrontation, stagnation; new treatment, diagnosis or therapist.	Desire to be a “good patient”	Fear of being seen as difficult or fear of losing the therapist.
Secondary circumstances	Fellow-patients who are noisy or rude, having to pay for coffee.	Awareness that one is using “scarce goods”	Not asking for more sessions because there is a long waiting list.

Articulate expressions (either assertive or adamant) were found in almost every post, but in different forms—and with different consequences. Patient articulation in mental healthcare develops over time and along different paths. Three stages could be identified: early, later and after therapy. An overview of the forms and stages we found is given in Table 3.

**Table 3:** Assertive and adamant behavior during different stages of therapy.

	Assertive	Adamant
1. Early stages of therapy: high distress, low (mental) health literacy.	Expression of needs, while respecting the other person and their expertise. “I have a deep desire to be taken care of.”	Making demands or expressing rights without respect for the other person and their expertise. “She earns from these sessions, doesn't she?”
2. Later stages of therapy: lower distress, increased (mental) health literacy.	Expression of needs, while respecting the boundaries of the dyad and taking responsibility. “She was there, with me, in the dark.”	Expressing needs or discontent, while stepping outside the dyad and/or not taking responsibility. “I have some wishes, which are actually demands.”
3. After therapy: either high or low distress, increased (mental) health literacy.	Contributing to the field of mental health care in a constructive way. “I tell my story and I see that it brings hope to other people.”	Attacking or dismissing mental health care as a whole, or their former therapist. “This is of course a kind of professional deformation induced by the compensation system.”

### 3.1 The assertive patient

#### 3.1.1 Early stages. “I have a deep desire to be taken care of”

When first entering mental health care, many patients find themselves in an unfamiliar world. They also experience high levels of distress and a deep, urgent need for relief. The combination sometimes leads to demanding behavior that, at first glance, resembles consumerist behavior, as in this patient's letter to her psychologist.

I am thinking of what I should tell you [about myself]. Preferably nothing, but you need information to do your job properly and help me. The only thing I want is the right treatment with a competent psychologist. [PB 01-01]

After having made the relationship almost transactional in nature, however, she proceeds to describe the underlying emotions:

But that's the thing, my moods are all over the place. I myself am not even keeping track anymore. On good days, I am talking a lot and feeling pretty confident. But on down days, I don't see how I will ever be cheerful again. I can't muster up putting a smile on my face, feel worthless and have no idea what I’m doing in life. Nothing about it seems fun anymore. I don't understand why people enjoy life. [PB 01-01]

She describes how she had some bad experiences and has lost trust in other people. Considering this, it seems unfair to label her earlier statement as consumerist. It seems a primary reaction to cope with the insecurities and discomfort of being in therapy. Similar accounts during the early stages of show patients feeling misunderstood, frustrated or powerless, not progressing as quickly as hoped for, having to adjust expectations, and fear of the therapists’ judgement. These feelings sometimes translate into sarcasm, condescension or distrust. Several patients recognize that their demanding behavior is part of the problem they want to solve:

I have a deep desire to be taken care of. (…) I know now it is my search for getting a bandage and a kiss, much like a parent does with a child. [PB 01-01]

These expressions can hardly be seen as a request to be seen as an adult and to decide, act and evaluate independently, as is the definition of articulate behavior. Patients were found to frequently look for leniency, sometimes even for the temporary exemption from the obligation to act as an adult altogether. This matches the attributions given by the therapists during this stage:

Some people really put forward demands and preconditions that we can't meet straight away. It makes the job tricky, but at the same time, tha's what we’re there for [at the department for personality disorders]. To work with that kind of people. But anyway, we try to find a balance. Not fully meet their demands but explore them (…) It becomes part of the therapy. [TI 005]

During this stage, there were numerous examples of psychologists understanding demanding or difficult behavior as a normal part of the therapeutic process and a component of the problem for which the patients seek help. They responded by assuring patients they were there for them, and helping them navigate the uncomfortable, scary initial stages of therapy. By employing a sort of “free pass” strategy, therapists help their patients, but also themselves, because they do not take the assertive utterings as personal criticism.

#### 3.1.2 Later stages. “She was there, with me, in the dark.”

Once therapist and patient are acquainted, many patients use assertive expressions. They do so by taking someone with them to a difficult conversation, explaining something about themselves, taking initiatives, negotiating, engaging in the therapeutic process, researching their disorder and possible solutions and discussing the outcomes, being open to tools and insights, persevering, proposing solutions, choosing to follow a therapist's suggestion when they agree with it, discussing difficulties and making requests.

Being ever purposeful, I often wondered (sometimes out loud during therapy) how we were going to tackle my “problem”. According to my psychologist, it was a matter of time. (…) I have often experienced impatience during therapy, a lot of despair too. But I can finally say it's working. [PB 01-03]

Assertions in this category were generally accompanied by expressions of reciprocity, self-regulation and perspective-taking. Patients expressed understanding for the therapist, were concerned about them or stood up for them. They approached them as a person, not as a commodity, and emphasized that they were an important factor in their lives. They analyzed the situation before responding and attempted to manage their emotions.

According to the patients, therapists respond to this type of assertions by apologizing if they made a mistake, facilitating the assertive response, promoting autonomy, setting boundaries, being open to suggestions by the patient, explaining, or following up with a question or intervention. In most cases this process resulted in a strong alliance between therapist and patient, either against the symptoms or against the system:

She was there, with me, on the other end of the line, calm and honest as always. (…) She said she knew how heavy it was. I knew she didn't only know, but understood through and through. (…) I realized at that moment she may have been closer than ever. In the dark, maybe even because of the dark. [PB 01-01]

When it comes to dealing with the system, therapists habitually spend vast amounts of energy to advocate and arrange things for their patients:

I can't find a place for her. This (…) has to do with [the complexity and comorbidity of] her symptoms: she used substances, so many options are eliminated. She has a developmental disorder, so fewer options still. So for weeks several people—her parents, us, the municipality—are on the lookout: (…) where can she have a time-out? But I just can't find it, it's tormenting me. [TI 003]

Assertive behavior during this stage of treatment doesn't seem to be problematic. It sometimes creates tensions, but it is based on reciprocity and both the interviews and the blogs indicate that it has the potential to enhance the relationship and the process. It is important to note that both patient and therapist stay within the therapeutic dyad and resolve issues inside the therapeutic relationship. They respect each other as (moral) equals.

This understanding of roles allows for flexibility, as in this example, where a patient was getting worse and unable to express her wishes and needs as clearly as usual. She was relieved her therapist organically took on a more active role in caring for her:

“She takes charge: clear questions, concerned looks and a firm advice (sleep medication immediately) follow. All my arguments not to take them are swept off the table, friendly but decisively.” [01-05]

#### 3.1.3 After therapy. “I tell my story and I see that it brings hope to other people.”

After therapy, assertive patients are more aware of the mechanisms behind, and discussions around, certain phenomena in the field. Their health literacy has increased and their distress often subsided. Most of them leave mental healthcare. However, when they are deeply moved by their experiences, they sometimes stay involved. They may join a patient organization or become peer counsellors.

My work as a peer counsellor gives me strength. You see what others go through. To help people through it with your own experiences, is healing to me too. I tell my story and I see that it brings hope to other people. [PB 01-05]

In their blogs, they often provide information about procedures and labels that patients are confronted with, or give advice about how to resolve issues during therapy.

### 3.2 The adamant patient

#### 3.2.1 Early stages. “She earns from these sessions, does't she?”

Although therapists are mostly patient and lenient with difficult behavior during the initial stages of therapy, particularly adamant behavior can put therapists on the defensive. This patient starts in a classic consumerist way:

“Glad I could come so quickly.” I cross my legs. Why am I saying this? Should I thank her on my knees for having me? She earns from these sessions, doesn't she? She asks what I expect from therapy. With the knowledge that I have to pay for the sessions myself in the back of my mind, I talk quickly: “My father killed himself, so I thought therapy would be a good idea. Just tell me what I should do to process this”. [PB 01-03]

This type of behavior made therapists feel uncomfortable or “commodified”:

These [insured] patients have to cough up a partial contribution. So it feels like I have to deliver. Some patients can really play on this (…) like: “you have to solve my problem because I’m paying you to. You are the psychologist. I present you my problems, you solve them.” I really would have liked to, but I can't. [TI 015]

Professionals found it difficult to respond to this type of interaction. As one experienced psychotherapist described:

People have been socialized differently than before. (…) Your authority is no longer recognized at face value. This is partly a good thing, you might say. But also… They can say: “I googled this and just do it”, or something like that. Like a comparative commodity research, in a distrustful way. “If you don't do this…” Like they are testing you. It happens a lot more often now than before, and you’re not as protected anymore. The customer may treat you disrespectfully, and you are like a sitting duck. [TI 014]

Several psychologists reported uncertainty when presented with this type of interaction:

I had several patients that made me wonder: do you want me to help you or not? If you think you know everything better, what are you here for? [TI 004]

The implicit rules of the therapeutic dyad are broken and therapists find themselves unable to do their work. One solution that therapists consistently used for this situation, was to apply the “free pass” strategy seen earlier, to give patients the benefit of the doubt because of their distress. This allowed them to reinstate the rules of the dyad and proceed with their work. Perhaps for this reason, therapists were remarkably reluctant to label behavior as consumerist or offensive, even when they experienced it as transgressive.

For example, I have been stalked for a while by a patient with borderline. (…) Very rigid, “cry for help”, but I had already explained to her several times: “I really can't do anything for you if you don't (…) accept a case manager from the [out-patient care team].’ She was wallowing in self-pity, there was also something wrong with her leg, so she always went “no, you guys have to come to me, I don't want to cycle 200 meters”. Those kinds of demands, “my way or the highway”. Like that. Clinging behavior, originating in the pathology. So it's a pattern in [these patients’] lives that they repeat the same behavior, since early childhood. Clinging everywhere to try to..well..get rid of their problem. [TI 024]

When exploring further whether he would consider *any* patient behavior consumerist, or adamant, he said no.

#### 3.2.2 Later stages. “I have some wishes, which are actually demands.”

In later stages, adamant behavior was sometimes observed when a patient was unhappy with the sessions, or had benefited from them but wanted to get more out of them. Like the assertive patient, the adamant patient expresses a right, a preference or an opinion, but the expression lacks awareness or shared responsibility for the therapeutic relationship, as if discussing a commodity or a product:

I have some wishes, which are actually demands. My therapist needs to be familiar with schema therapy, be willing to support instead of treat (with the goal of getting “better”), and the most important thing, I should not have to be “finished” within a year. I want to be able to stay as long as I feel the need. [PB 02-01]

Sometimes, the therapist's motives are questioned, as in this fragment, where the therapist suggests referring the patient to another provider, who might have a shorter waiting list for a specific treatment:

Luckily I was well-informed about the actual situation. I was upset by the fact that she was not telling me the truth about the availability of this specific treatment. On the spot I realized that the psychologist tried to get rid of me with incorrect information. I expressed this feeling, considerably irritated by the stated falsehoods. [PB 01-03]

One woman was unhappy that most of her therapists were absent during the summer holidays:

After all, I can hardly postpone my problems and sorrows until my therapists return well-rested from their holiday destinations. [PB 02-02]

Patients describe taking charge to set the record straight and question the usefulness of diagnoses or treatments. Their statements are in stark contrast with the excerpts from the *Assertive* category. They lack internal processes such as perspective taking, and the therapist as a person is largely absent. Therapists have become a means towards an end. The response of the therapist to the patient's statement is not always described in the blogs. When it is included in the narrative, the response is often negative or defensive.

The therapists’ accounts provide an answer as to why that is. Therapists feel that adamant patients turn the tables on them, and they have insufficient means to defend themselves:

It increases your vulnerability, because these weapons are validated, right? (…) Yes, it is validated more to have an attitude or treat you like… they are allowed to make those demands. And they are allowed to act more out of line than is decent. (…) And it’s more difficult because people take ownership of this attitude and are reassured that it’s allowed. [TI 014]

This type of interaction also alerts therapists to the possibility that the disagreement may turn legal. This experienced clinical psychologist describes his interactions with a 17 year old girl and her family:

She was so depressed that I thought: medication would be the best option. But they decidedly said no, nor did they want to take her to a specialized facility. But I can't withhold care, right? Once you said “yes” you’re [legally] obliged to carry the treatment through. (…) I feel like I’m being pressured by her parents. (…) They blame me when, in their eyes, progress is not optimal. I feel pressured. The pressure of unhappy parents. It made me think: I hope it doesn't turn into a complaint. I feel vulnerable. [TI 010]

When this type of interaction can't be resolved therapists adopt a defensive attitude, referring to goals in the treatment plan (agreed on with the patient), or resorting to rules or protocols. These are legitimate actions to protect the quality of the treatment, but in this context, they serve as protection for the therapist too. Something which makes patients, in turn, feel vulnerable.

I know I am not the ideal patient (…). So sure, whatever you want. If you guys believe in this [evaluation]. I really don’t care for such a scholastic approach, but let’s get on with it. (…) The good news is that during my last evaluation, things got so out of hand, with so many reproaches back and forth, that it dawned on me that I really needed to take myself elsewhere. (…) I am now finally with a good therapist, who doesn’t do evaluations. [PB 01-01]

In most cases, therapists and patients described that it was difficult to backtrack once this route was taken. Re-establishing trust and reciprocity requires the utmost from both patient and therapist in terms of emotional and communicative skills.

#### 3.2.3 After therapy. “This is of course a kind of professional deformation induced by the compensation system.”

Some former patients, mostly those who had negative experiences, become activists against (what they consider to be) “incompetent” professionals after treatment. They now understand the system better and may want to protect others from the same experience, for instance by calling out problems.

[I think mental health care professionals] often lack expertise on giftedness and that they are always looking for a DSM diagnosis to explain symptoms. This is of course a kind of professional deformation induced by the compensation system, but doesn't do justice to the person and the personal story. [PB 03-05]

Like their assertive counterparts, adamant patients find ways to contribute, but in a militant way leaving little room for dialogue.

Don't economize youth mental health care. Don't let a municipal official decide on the future of your cousin, your daughter, your neighbour. Anyone can suffer from a psychological illness. [PB 03-01]

They may interview and warn other patients, write manifestos about what they consider to be mistreatment, approach professionals on social media, or start to provide services themselves, as peer counsellors.

Others re-enter health care with a different attitude.

Through a hospitalization at facility X (where I was casually misdiagnosed) I had them carry out an assessment to get my (old) diagnosis back and my medication. [PB 02-05]

Still others file complaints and lawsuits against their former therapists. These actions trickle into the consultation rooms. Many therapists felt that when a dispute is taken outside of the therapeutic dyad, anything can happen.

I feel increasingly vulnerable. (..) Even if you acted with the best of intentions. Because the general tendency is towards accountability, and all complaints must be honoured. Or well, examined. I feel more vulnerable. Fair game. [TI 010]

Even if therapists feel confident that they would be able to defend their actions, they find it conceivable that an outside bureaucrat might find fault. Hence they rely on files, rules and protocols to defend themselves, but these are so complex, vast and sometimes contradictory, that professionals hardly consider them helpful.

I am well aware that we make mistakes. People are allowed to complain. (…) It's a good thing, that people have that right. But it scares me sometimes too. I go back in my mind and wonder: have I ticked all the boxes? How “wrong” could I be if someone checks everything? [TI 015]

For therapists who had faced a lawsuit or complaint, the process was draining, scary and long. One respondent, who was investigated after a patient's suicide, described the experience as traumatic. Several respondents mentioned acting defensively because they were aware of the possibility of a complaint or law suit, for instance by going along with the patient's demands to avoid difficulty.

## 4 Conclusion

Taking these limitations into account we found that the logic of choice or consumerism takes a particular shape in mental healthcare. An important distinction is made between assertive and adamant behavior. Assertive patients, befitting the logic of care, express wishes and needs, but they do so with respect for therapists, their expertise and the unwritten rules of the therapeutic dyad. They take responsibility for their own role in the therapeutic process. Of course, the early stages of therapy are scary—health literacy is low and distress is high. For these reasons patients can count on almost endless patience and support from their therapists. From the middle stages of therapy onwards, most patients and therapists have aligned their expectations and find a mode to navigate difficult moments and decisions together. This alliance allows for role flexibility: when the patient's life, symptoms or circumstances take an unexpected turn and they are unable to act as equal participants who decide, act and evaluate independently, patients can let go and allow their therapist to take care of them.

When articulate behavior takes a more adamant form, however, befitting the logic of choice, the dynamic plays out differently. The adamant patient is not open to discussion, puts forward high demands from the start, and sometimes outright forbids their therapist to have an opinion of their own. These patients initially receive the same leeway as anyone, as it is understood that their behavior may originate from their pathology, distress or health illiteracy, and that empathy will contribute to a stronger working alliance. But therapists do feel more irked and guarded with these patients, keep the possibility of a complaint or lawsuit in the back of their mind and are more likely to act defensively. Professionals become unsure of how to behave. The very “commodity” the patient is looking to obtain, the therapeutic relationship, is damaged.

Therapists are trained to mobilize the therapeutic relationship to move towards treatment goals. This includes handling difficulties or ruptures in the alliance. The interactions in the assertive category consistently show that therapists apply this aspect of their training to clinical practice, and that their efforts are received well by patients. However, that training does not seem to be sufficient to deal with particularly consumerist or overly articulate behavior, merged and renamed in this study as adamancy. The adamant patient appears not that interested in the relationship with the therapist as a moral equal, has a more transactional approach and has limited motivation to use the relationship as an opportunity to explore their own interactional patterns. The adamant patient, coming from the logic of choice, will argue that problems they experience with the therapist are not in the interaction, but in the therapist who is not “delivering”.

## 5 Limitations

Studying blogs comes with a number of limitations. A search engine like Google might have a different rationale in terms of relevance than the researchers. Relevant content may have been missed. Blog moderators might have an agenda in promoting or withholding specific narratives. Researching online narratives also excludes those mental health care users who don't have access to devices, or who lack digital knowledge and skills (44). So, the sample will necessarily be limited to a subset of patients with specific characteristics. For instance, so-called e-patients are more likely to be young and to take an active role in managing their own care (45, 46).

Qualitative interview studies are almost always small-scale and the same goes for ours, based on 25 interviews with therapists. In addition: building a theory bottom-up—constructing stages and types of articulate behavior—is necessarily somewhat speculative. Deductive research is needed to corroborate the stages and distinctions found in this study.

## 6 Recommendations

These findings call for a few recommendations for mental health care. A first recommendation would be to train therapists to catch patients’ concerns early and thereby reduce the odds of adamant behavior developing. Therapists are already aware of the prompts and obstacles (diagnoses, protocols, transferring a patient to another therapist or department when necessary) inherent to the therapeutic environment that could trigger a response in the patient, but judging by the patients’ accounts, sometimes therapists still miss signs that their patient is overwhelmed or confused. The results from this study suggest that therapists would be well advised to realize that many patients don't (immediately) know how to feel or react under these circumstances, and that checking back with them would be helpful.

Additionally, therapists might try to teach patients to remain in the assertive side of our table. Buetow's (47, 48) person-centered framework might be helpful here, as an alternative to the “golden standard” of client- or patient-centered care inherent to many health care systems. In Buetow's opinion the patient is not excused from relational norms because the relationship is “professional” or they are “too sick”. Buetow argues that “patienthood can mask personhood”, and that this can be unhelpful and dehumanizing. He proposes, for instance, to reinforce patients to engage in their internalized norm of helping others (their therapists) in return for their help. Caring behavior could be to express gratitude, acknowledge therapists’ concerns and fears, forgive therapists for their mistakes, to express regret when making a mistake themselves, and to contribute to health research and provider education. These are behaviors that many patients in the *assertive* category display already. Therapists and their colleagues (receptionists, assistants) can induce and coach this behavior further. Buetow does recognize that there could be situations in which patients are, temporarily or permanently, genuinely restricted in their ability to care. But he sees this as rare exceptions instead of a common occurrence, as the therapists in this study appear to do. His viewpoint could help therapists regain agency in situations where they feel uncertain how to comprehend the interaction and respond appropriately.

A second recommendation could be taken up by organizations. Many examples can be found in the literature of what organizations can do to foster a relationship-centered culture (49, 50) or, in our terminology, to prevent adamancy. Organizations could invest in promoting caring behavior and inhibiting unnecessarily adamant behavior in patients, for instance by reconsidering putting patients in a “customer” position. For example, many waiting rooms in mental health organizations are decorated with posters outlining the procedure to file a complaint, requests to engage in activities associated with accountability and customer satisfaction, such as Routine Outcome Monitoring, instructions to check with the receptionist if they have to wait for longer than ten minutes, and (in the Netherlands) appeals to rate the institution on a national website devoted to the evaluation of health care providers. Instead, material could be provided that requires patients to reflect on and emotionally invest in relationships of mutual care, such as posters with suggestions for reciprocal polite, caring behavior.

A third recommendation would be directed at society at large or policy makers. The move toward consumerism or the logic of choice in mental healthcare and elsewhere sprouts from societal beliefs and policy choices. Reconsidering its appropriateness could also be seen as a collective endeavor [e.g., (51, 52)]. With regard to mental healthcare, it would be especially important to realize that adhering to the logic of choice is not only unbecoming or unsuited but actually a threat to the relationship between patient and therapist that is key to treatment success. The consultation room should be a safe space, where patients discuss their problems openly but respectfully, and where therapists feel free to give care without fear.

## Data Availability

Only part of the dataset presented in this article is readily available. The interview data are not readily available due to patient privacy and sensitive information. The written narratives are available upon request. Requests to access the datasets should be directed to LKM, linda.mulders@uvh.nl.
